# Chromatoid Bodies in the Regulation of Spermatogenesis: Novel Role of GRTH

**DOI:** 10.3390/cells11040613

**Published:** 2022-02-10

**Authors:** Rajakumar Anbazhagan, Raghuveer Kavarthapu, Maria L. Dufau

**Affiliations:** Section on Molecular Endocrinology, Division of Developmental Biology, Eunice Kennedy Shriver National Institute of Child Health and Human Development, National Institutes of Health, Bethesda, MD 20892, USA; anbazhagan@nih.gov (R.A.); raghu.kavarthapu@nih.gov (R.K.)

**Keywords:** phospho-GRTH, chromatoid bodies, spermatogenesis, transcriptome analysis, RNA transport, RNA storage

## Abstract

Post-transcriptional and translational control of specialized genes play a critical role in the progression of spermatogenesis. During the early stages, mRNAs are actively transcribed and stored, temporarily bound to RNA binding proteins in chromatoid bodies (CBs). CBs are membrane-less dynamic organelles which serve as storehouses and processing centers of mRNAs awaiting translation during later stages of spermatogenesis. These CBs can also regulate the stability of mRNAs to secure the correct timing of protein expression at different stages of sperm formation. Gonadotropin-regulated testicular RNA helicase (GRTH/DDX25) is an essential regulator of spermatogenesis. GRTH transports mRNAs from the nucleus to the cytoplasm and phospho-GRTH transports mRNAs from the cytoplasm to the CBs. During spermiogenesis, there is precise control of mRNAs transported by GRTH from and to the CBs, directing the timing of translation of critical proteins which are involved in spermatid elongation and acrosomal development, resulting in functional sperm formation. This chapter presents our current knowledge on the role of GRTH, phospho-GRTH and CBs in the control of spermiogenesis. In addition, it covers the components of CBs compared to those of stress granules and P-bodies.

## 1. Introduction

Spermatogenesis is a complex, serial and highly specialized differentiation program during which progenitor cells undergo a series of cellular reorganization events, resulting in the production of mature functional sperm [1,2,3]. This differentiation process is controlled by the integrated expression of an array of genes and specific proteins in a precise temporal sequence that produces genetically unique spermatozoa [2,3,4]. Gene expression in haploid spermatids requires temporal uncoupling of transcription and translation in the adult mammalian testis [2,5,6]. Post-meiotic haploid round spermatids formed during the spermatogenesis process possess complex transcriptomes, and hence efficient and accurate quality control mechanisms are necessary to deal with the major diversity of transcribed RNAs in these germ cells [7]. Initial stages of spermatogenesis are marked by active transcription and the resulting mRNAs are transported and stored transiently in large cytoplasmic ribonucleoprotein granules called “chromatoid bodies” (CBs) [8,9].

The CB is a perinuclear organelle and a ribonucleoprotein (RNP) granule present in the cytoplasm of male germ cells [9]. The functions of CBs overlap with those of P-bodies and stress granules of somatic cells. Due to the presence of a wide array of proteins involved in different steps of RNA metabolism with different classes of RNAs, including microRNAs (miRNAs) and Piwi-interacting RNAs (piRNAs), the CB seems to function as an RNA processing center [9,10]. CBs are involved in diverse pathways and mechanisms through which it regulates a wide variety of processes, including RNA transport, regulation, decay, surveillance, translational arrest, translation machinery assembly, etc. Although interesting aspects have been found in the last half-decade, several intrinsic mechanisms (specifically, the role of CBs in the storage and regulation of important mRNAs that are involved in making healthy sperm) are still not fully understood in detail [10]. Mature mRNAs are transported to CBs during the early stages of spermatogenesis (active transcription stages) bound to RNA binding/transport proteins such as gonadotropin-regulated testicular RNA helicase (GRTH/DDX25) [9,11,12].

## 2. RNPs Are Critical for Cellular and Organism Function: Role of CBs

RNP complexes and granules are powerful composite structures of merged functions and unique properties. RNP granules are formed under physiological conditions. In addition, granule formation can be induced by stress such as heat, starvation, oxidative stress, etc. These granules are more dynamic in nature, and they occur in condensed or diffused states and depend on the cell requirements. Specific mutations occurring in the cell can prevent RNP assembly or disassembly and diminish cellular and organism function, resulting in several pathologies. Examples include fragile X-syndrome, associated primary ovarian insufficiency in human germ cells, and amyotrophic lateral sclerosis (a progressive neurodegenerative disease that affects nerve cells in the brain and the spinal cord), possibly occurring due to the failure of disassembly of stress granules. The importance of RNPs is diverse and the complete functions are still not completely understood. The main functions of RNPs include carrying out intricate tasks in RNA-processing pathways and the post-transcriptional regulation of mRNAs. The association of RNA-binding proteins such as DDX4 and RNA in the germ line of several organisms gives rise to non-membranous structures called germ granules. These are known by several different names in different organisms with minor variations in function, from Nuage, Pole plasm, piNG body (piRNA nuage giant body) in *Drosophila,* Polar granule in *C elegans,* and chromatoid body, Balbiani body, and Cajal body in mice and zebrafish [13]. One of the biggest RNP granules is the CB, which consists primarily of RNA and other RNPs involved in the RNA processing of male germ cells.

The CB is a membrane-less, filamentous-lobular, perinuclear organelle (0.5–1 μm) and an electron-dense structure present in the cytoplasm of male germ cells [9,14]. It acts as a repository of important mRNAs associated as mRNPs waiting for translation during later stages of spermatogenesis. CBs dynamically move within the cytoplasm and exhibit continuous changes in shape and size. The biochemical composition of CBs is unique: they are primarily composed of small RNAs, Piwi-interacting RNA (piRNA), siRNAs, miRNAs, mRNAs, long non-coding RNAs, RNA-binding proteins, members of the small interfering RNA pathway such as MIWI, Argonaute protein, Dicer endonuclease, decapping enzyme, and other proteins involved in RNA post-transcriptional regulation which may play a critical role during sperm elongation and spermiogenesis [8,9,15,16]. CBs also contain GRTH [9], phospho-GRTH [10] and MVH/DDX4, a mouse homolog of VASA, a germ cell marker [16]. In fact, these proteins constitute the majority of CBs in addition to other proteins which are involved in the piRNA pathway, nonsense-mediated RNA decay (NMD) pathway, and in RNA post-transcriptional and translational regulation. Specifically, these CB proteins are DDX25, DDX4, MILI, MIWI, TDRD6, TDRD7, D1PAS1, PABP1, HSPA2, and constitute around 70% of the CB proteome [9,10,17,18].

**Figure 1:**
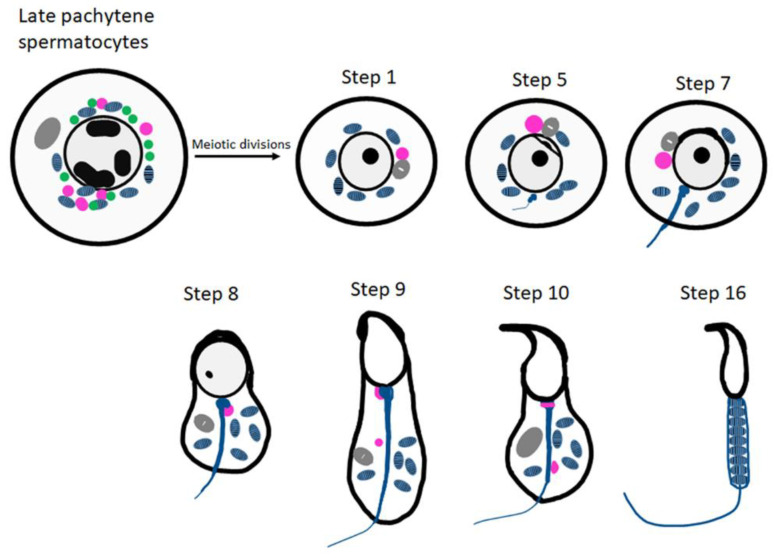
**Schematic representation of CB organization and fate during spermiogenesis in mice.** The inter-mitochondrial cement (IMC; green) intermixed with mitochondria (blue) and the CB precursors (pink) co-exist in late pachytene spermatocytes. The Golgi complex is depicted in gray. The CB (pink) is condensed to its final single form in the early round spermatids. At step 8 of spermiogenesis, the CB is found at the basis of the flagellum. Later, it splits into two separate structures (step 9 onwards) and eventually disappears.

The origin of CBs is highly debatable. The most accepted view is that they emerged from small granules (precursors of CBs) which are associated with the nuclear envelope present near the electron-dense inter-mitochondrial cement in the late pachytene spermatocytes (Figure 1). The CB can be detected during all steps of round spermatid differentiation (steps 1–8 of spermiogenesis). The largest CBs are observed in step four, five and six of spermiogenesis [18]. At later stages of spermatid elongation, CBs move caudally to the neck region and split into two separate structures; one is discarded along with residual cytoplasm, and the other forms a ring around the base of the flagellum (Figure 1).

There is limited information about the specific mechanisms of CB function during spermatid elongation. With the identification of small non-coding RNA-mediated gene regulation and other associated mechanisms, the functions of the CB are slowly beginning to be understood. However, several of the molecular mechanisms are still unclear, requiring further in-depth molecular studies.

## 3. CBs Are Analogous to Stress Granules and P-Bodies

Compartmentalization of molecular processes is accomplished by various intracellular organelles that spatially segregate functionally related molecules. RNPs act as organelles that lack any demarcating membrane and play a key role in mRNA homeostasis. RNP granules formed under physiological conditions in male germ cells are called CBs, while in somatic cells they are called RNA processing bodies or P-bodies. CBs’ overall functions fall between P-bodies and stress granules and are in concert with the maintenance of RNA regulation. Stress granules and processing bodies are also membrane-less RNA granules that dynamically sequester translationally inactive messenger ribonucleoprotein particles (mRNPs) into compartments which are distinct from the surrounding cytoplasm [19,20]. These granules are more dynamic in nature and exist in a condensed or diffused state based on conditions and requirements. Like P-bodies, stress granule assembly is dependent on the pool of non-translating mRNAs. Stress granules and P-bodies can physically interact to facilitate the shuttling of RNA and protein between them. The main difference between P-bodies and stress granules is that P-bodies assemble around the key enzymes of cytoplasmic RNA degradation in physiological conditions, and stress granules assemble around the essential components of the translation machinery under different stress conditions such as heat, glucose deprivation, viral or bacterial infection, hypoxia, and oxidative stress [19,21].

**Figure 2:**
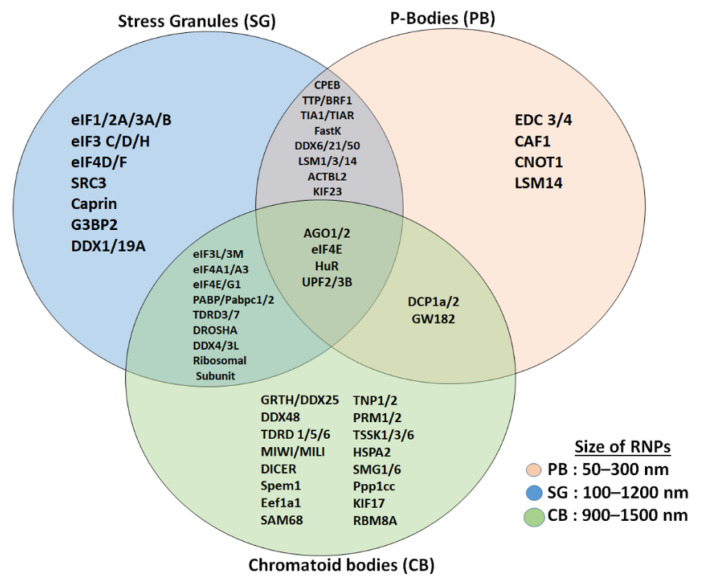
**Comparison of proteins (mRNA decay and translation machinery) present in CBs with stress granules and P-bodies.** Stress granules and CBs share several initiation factors and translation initiation assembly proteins, while P-bodies and CBs share few of the proteins. CBs are more related to stress granules than to P-bodies and CBs are bigger in size compared to stress granules and P-bodies.

In contrast to CBs and stress granules, P-bodies are not associated with the regulation of translation initiation; instead they serve as a site for mRNA degradation, translation repression, storage of non-translating mRNAs, and RNA-binding proteins (Figure 2) [10,20,22]. P-bodies are uniquely enriched with factors related to mRNA decay and the NMD pathway, such as members of mRNA decapping machinery including the decapping enzymes DCP1/2; UPF1/2, the activators of decapping EDC3, Dhh1/RCK/p54, Pat1, Scd6/RAP55, and EDC3; the LSM1-7 complex; and the exonuclease XRN1 (Figure 2) [20,22,23,24]. P-bodies are independent of initiation factors or translational assembly, while CBs seem to regulate mRNA storage and decay, as well as translational machinery, all in one compartment effectively [10,20]. Recent studies have shown that the process of liquid–liquid phase separation is a main driver promoting the assembly of RNP granules such as stress granules, P-bodies, and other related membrane-less organelles [25]. It would be very interesting to determine the involvement of biological mediators and other proteins in the assembly of RNP granules. Few studies have implicated post-translational modifications of RNP granule proteins as the driver for the assembly and disassembly of the RNP granule [13,19]. Irrespective of the exact driver which initiates the formation of the RNPs, the role it plays is unique and irreplaceable, and has a critical role in the survival of the cell. More like stress granules, CBs have proteins or mRNA that are involved in the translation process, such as initiation and elongation factors, ribosomal subunits and other associated factors, and mRNA regulatory factors such as PABPC1, UPF2, and others [10,17,21]. RNP granules induced by heat stress are detected in spermatogonia, preleptotene, and early pachytene spermatocytes [26]. Detailed information and similarities of these heat-induced RNP granules in germ cells with respect to CBs are not clear. Further studies on interaction between these RNPs inside the cell provide vital clues on the molecular mechanisms of gene regulation. CBs resemble the recently described TIS-associated granules and the interconnections proposed between TIS granules and the ER (TIGER domain structure) [27].

## 4. Small RNAs of CBs and Regulation of Spermatogenesis

During spermatogenesis, the early precursors of sperm utilize small regulatory RNAs such as microRNAs to control the expression of an array of genes at transcriptional or post-transcriptional levels during complex and specialized processes of sperm production. The role of CBs in small RNA-mediated gene control and the associated mechanisms are not clearly delineated at present. The repertoires of microRNAs and piRNAs have been identified and both serve as important regulators of male germ cell differentiation.

miRNAs are small in size (21–23 nucleotides) and belong to the class of noncoding RNAs which act as endogenous gene regulators and participate in a wide array of biological functions, by promoting target mRNA degradation and inhibition of translation [28,29,30]. Each miRNA can target several mRNAs of different genes and thus regulates gene expression stringently in a stage- and tissue-specific manner in every organ including the testis. miRNAs recognize their target mRNAs by sequence-specific bp pairing in the RNA-induced silencing complex together with Argonaute proteins (AGO) [9]. Most miRNAs are derived from primary miRNA transcripts which are processed by the Drosha-DGCR8 complex in the nucleus to generate precursor miRNAs (pre-miRNAs), which are transported to the cytoplasm where mature miRNAs are generated via a Dicer-dependent or independent route. One of the crucial components of the miRNA and siRNA pathways is the cytoplasmic endonuclease Dicer, which is critical for male fertility [31]. Dicer interacts with the germ-cell-specific RNA helicase MVH (mouse VASA homolog). Sertoli cell-specific deletion of Dicer in mice results in spermatogenic malfunction, defective maturation, and infertility [31]. Transcripts of AGO proteins, Drosha, and Dicer have been demonstrated to be present in germ cells and Sertoli cells [29,32]. GRTH regulates proteins of the microprocessor complex, Drosha and DGCR8 (miRNA biogenesis) at the mRNA and protein levels [29]. miRNA pathway proteins have been demonstrated to accumulate in the CBs in haploid round spermatids, suggesting that the CB and GRTH have a role in miRNA-dependent gene regulation [28,29].

Testis-specific miRNAs such as miR-469, testis-preferred miR-34c, miR-470 and others such as let-7 family members (let-7a/d, b, and e-g), and miR203 were upregulated in round spermatids of GRTH KO mice. Furthermore, the enzyme complex (Drosha-DGCR8) required for the processing of miRNA was also upregulated in the KO mice. MiR-469 target TP2 and Prm2 mRNA by binding to their coding regions and thereby preventing translation of these essential mRNAs to proteins [29]. GRTH negatively regulates overall miRNA biogenesis via Drosha/DGCR8 microprocessor complex, to generate mature miR-469 and others which could play a role during spermatogenesis. miRNAs such as miR-469, through their inhibitory action on TP2/Prm2 mRNA, control the timely expression for chromatin compaction and the progression of spermatogenesis [29].

piRNAs comprise the biggest and most complex class of small non-coding RNAs. Unlike miRNAs (22 nt long), piRNAs are slightly longer, in the range of 26–32 nucleotides, and the biosynthesis of piRNAs is different from that of miRNAs and siRNAs [33,34]. During post-meiotic germ cell differentiation, the CB accumulates piRNAs and proteins of piRNA machinery, as well as several other proteins involved in distinct RNA regulation pathways. The embryonic and post-natal male germ cells express high levels of piRNAs during late meiotic cells and haploid round spermatids. CBs provide platforms for the Piwi-interacting RNA (piRNA) pathway and appear to be involved both in piRNA biogenesis and piRNA-targeted RNA degradation. In addition, RNA regulatory mechanisms, such as the NMD pathway, are also known to exist inside the CB, and this provides exciting new insights into the function of CBs. Another important and most fascinating feature of the CB is its dynamic and non-random movements in the cytoplasm of haploid spermatids, thereby facilitating the sharing of selective mRNAs, small RNAs and proteins between neighboring spermatids, and making them an attractive organelle for specific pathways’ interconnectivity, in addition to coordinating mRNA regulation in an efficient and accurate manner.

## 5. GRTH-Mediated Regulation of Germ-Cell-Specific mRNAs in CBs during Spermatogenesis

**Figure 3:**
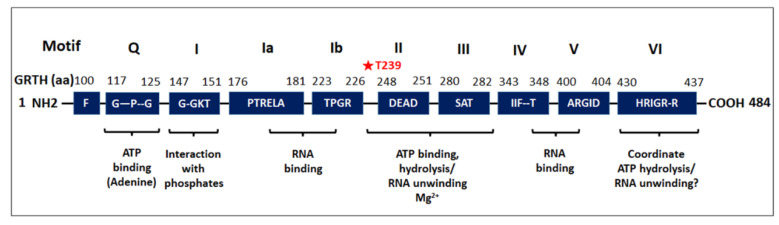
**Schematic representation of the conserved motifs of the DEAD-box family of RNA helicase present in GRTH.** Nine conserved motifs of GRTH protein were indicated as Q, I, Ia, Ib, II-VI. Amino acid positions of GRTH corresponding to each conserved motif are indicated on top of each motif. Aside from the conserved motifs, GRTH displays low amino acid similarity with other DEAD-box family of RNA helicases. Amino acids within the conserved motifs are specified by their letter code. Among the several serine and threonine residues in the GRTH, only T239 is phosphorylated by PKA (which is highlighted by a red asterisk).

GRTH, first identified in our laboratory, is a multifunctional RNA helicase and is a member of the Glu-Asp-Ala-Glu (DEAD)-Box family of proteins which plays an essential role in the process of spermatogenesis [35]. GRTH displays low amino acid sequence similarity with other members of the DEAD-box protein family (Figure 3). It is expressed in meiotic spermatocytes, round spermatids, and Leydig cells, and its expression is controlled by hormonal stimulation via gonadotropin/androgen regulation [35,36].

GRTH acts as a negative regulator of steroidogenesis (in Leydig cells) and mitochondrial, death receptor, and nuclear factor k-B (NF-kB) pathways, and plays a central role in preventing germ cell apoptosis [37]. In addition to its inherent helicase activity, GRTH transports mRNAs from nucleus to cytoplasm and to the CBs, and has a vital role in the completion of spermatogenesis. GRTH binds to specific mRNAs as an integral component of RNPs [38]. Furthermore, GRTH is also associated with polyribosomes to regulate target gene translation during germ cell differentiation [39]. The high transcriptional activity in early round spermatids, and mRNA transported by GRTH and its subsequent storage in CBs, provide critical control mechanisms to participate in the post-transcriptional RNA regulation.

GRTH knock-out mice are sterile with halted spermatogenesis (step eight of round spermatids stage) with no elongated spermatids, and the sperm and lumen of the epididymis contain only degenerating spermatids. These mice have normal levels of circulating gonadotropins and testosterone with normal sexual behavior. The CBs of GRTH KO mice are highly condensed and markedly reduced in size, with a lack of the “nuage” appearance which is usual at all steps of round spermatids. These changes in the CB of null mice are consistent with their lack of GRTH-dependent nuclear-cytoplasmic transport of messages concerned with the progress of spermatogenesis. Microarray differential gene expression analysis of polysome-bound RNA revealed a genome-wide perspective of GRTH-regulated genes, with the ubiquitin-proteasome-heat shock protein signaling network pathway and NFkB/TP53/TGFB1 signaling networks [39].

**Figure 4:**
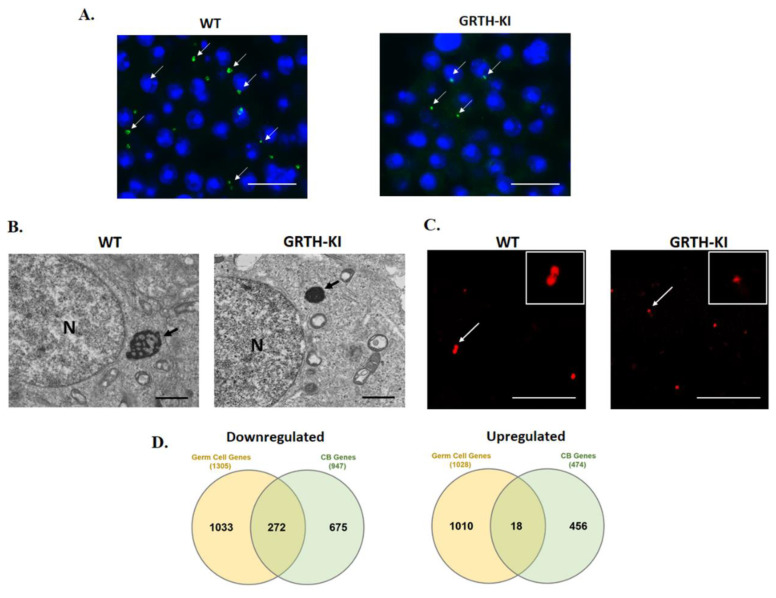
**Cellular and transcriptomic changes occurring in CBs of GRTH-KI mice compared to wild-type mice.** (**A**) Immunofluorescence staining of MVH/DDX4 (green) in the testicular tissue sections of WT and KI mice, CBs indicated by arrow; scale bar: ~25 μm [10]. (**B**) EM images from testicular sections with round spermatids (RS) showing a lobular structure with an irregular network of the less dense strands characteristic of the CB clearly visible in WT, while in GRTH-KI, CB is markedly reduced in size. Arrows indicate CBs and N-Nucleus; scale bar: ~1 μm [10]. (**C**) Immunofluorescence staining of MVH/DDX4 as a marker showing CBs (red) isolated from GRTH-KI mice are smaller in size compared to WT. Inset shows detail of CBs indicated by arrow; scale bar: ~25 μm [10]. (**D**) Comparison of gene expression of downregulated/upregulated genes in germ cells with a decrease/increase in the abundance of genes in CB obtained from wild-type and GRTH-KI mice [10]. Detailed methodology on immunofluorescence, EM, and RNA-Seq analysis were described earlier [10].

In germ cells, there are two species of GRTH: a 56 kDa non-phosphorylated form, predominantly found in the nucleus; and the 61 kDa phosphorylated form (pGRTH), present exclusively in the cytosol and found to be associated with polyribosomes. Previous studies from our laboratory have shown that 5.8% of Japanese infertile men (non-obstructive azoospermia) have a specific missense mutation (R^242^H) in the gene expressing GRTH, which resulted in the lack of phospho-GRTH (pGRTH). GRTH knock-in (KI) transgenic mice (human mutant GRTH gene with R^242^H) lack the 61 kDa phospho-species in CBs and cytoplasm, while the non-phospho form is still present in the cytoplasm, nucleus, and CBs of germ cells [4,10]. The levels of androgen and gonadotropin were not altered, and the mating behavior was normal. GRTH KI mice (lacking phospho-GRTH) are sterile, with reduced testis size and complete lack of sperm and elongated spermatids due to spermatogenic arrest at step eight of round spermatids. The round spermatids of GRTH KI mice contain CBs which are significantly smaller in size and more condensed compared to the CBs of WT mice (Figure 4A–C). 

The absence of the phospho form of GRTH in the CB of GRTH KI mice has direct impact on the structural integrity of CBs in RS, as phospho-GRTH is one of the abundant RNA binding proteins along with other CB proteins such as MVH, MIWI, etc. Previous studies from our laboratory demonstrated that GRTH protein, through its conserved binding motifs, bind to the 3′ UTR region of Tp2 mRNAs [40]. In a recent study, we also demonstrated that it is the phospho form of GRTH which has a more important role in the translation of Tp2 through the binding of its 3′ UTR [4]. In round spermatids, GRTH participates in the transport of specific mRNAs from the nucleus to cytoplasmic sites via the CRM1 pathway. In the cytoplasm, GRTH is phosphorylated and this phospho-GRTH association with relevant messages prevents their degradation, and presumably participates in the transport of mRNA to chromatoid bodies (CBs) where messages are temporarily stored and translationally repressed, awaiting translation during later stages of spermiogenesis [6,9].

The loss of phospho-GRTH in GRTH KI mice serves as a very good model to study the relevance of GRTH phosphorylation in relation to CB transcriptomic/mRNA storage profiles and healthy sperm formation. The CBs obtained from the GRTH KI mice were markedly smaller in size compared to WT mice which were analyzed using electron microscopy (Figure 4B). Furthermore, amorphous “nuage” texture with irregular boundaries which is typical for CBs were lost completely.

The CBs of mutant mice germ cells are small in size and store very few mRNAs which are essential for sperm formation in addition to the marked decrease in half-lives of mRNAs. The levels of non-phospho GRTH were unaltered, while pGRTH protein was completely absent in the CBs of GRTH KI. This model clearly illustrates the functions mediated by phospho-GRTH in round spermatids.

**Figure 5:**
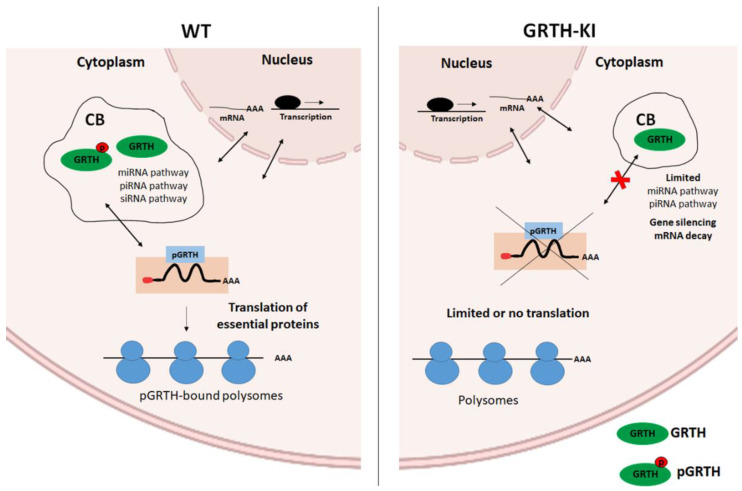
**RNA transport and associated changes occurring in CBs of GRTH-KI mice compared to wild-type mice.** GRTH transports essential mRNAs from nucleus to the cytoplasm for translation. The pGRTH, through its interaction with actively elongating polyribosomes, regulates translation of target mRNA. It is involved in transport of mRNPs in and out of the chromatoid body, where it is transiently stored and/or degraded when not needed in the CBs. mRNAs are transported from the CB by pGRTH for translation at specific times during spermatogenesis.

Phospho-GRTH is one of the important structural proteins of CBs [10] along with MVH and MIWI. In GRTH KI mice, the levels of MVH and MIWI were not altered; however, phospho-GRTH was completely lost. mRNA transport to the CB decreased due to the loss of phospho-GRTH, resulting in the diminished size of CBs in the GRTH-KI mice (Figure 4 and Figure 5). 

Furthermore, GRTH protein binding to specific germ cell mRNAs (Tnp1/2, Prm1/2, Grth, and Tssk6) in the CBs was highly diminished in the absence of phospho-GRTH in GRTH KI mice. This also clearly shows that the GRTH binding to important mRNAs is the phospho-form of GRTH.

**Figure 6:**
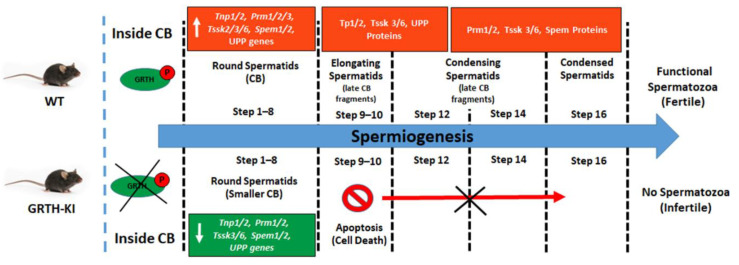
**Schematic diagram showing progression of the spermatogenesis process in the presence and absence of pGRTH, with reference to chromatoid bodies and the associated mRNA and protein expression.** Loss of phospho-GRTH altered the CB structure and biochemical composition. It also diminished the transport of essential transcripts between cytoplasm and CBs, thereby altering the mRNA storage profiles and causing impaired spermatid elongation, resulting in loss of spermatozoa and subsequently infertility.

Since the mRNA binding function in the CBs was impacted significantly in GRTH KI mice (lacking pGRTH), this resulted in impaired chromatin compaction and spermatid elongation, and stalled spermiogenesis at step eight due to the failure of the translation machinery assembly and the loss of mRNAs to decay inside the CBs (Figure 6). The transcriptome profiles of germ cells are exceptionally diverse and RNA-Seq profiles of isolated CBs (mRNA storage profiles) show altered expression of mRNAs involved in spermatid development, differentiation, chromatin remodeling, RNA transport, and transcriptional and translational regulation [10]. A comparison of gene expression in germ cells, with changes in abundance of genes in CBs obtained from wild-type and GRTH-KI mice, is depicted in Figure 4D.

During the process of spermiogenesis, the RS undergoes the elongation process where the histones are hyperacetylated and replaced by the highly basic transition proteins TNP1/2, which constitute 90% of the chromatin basic proteins, followed by the deposition of protamine PRM1/2 [41,42]. These chromatin remodeling proteins play a crucial role in hyper-chromatin condensation and compaction of the RS nucleus and reshaping the nucleus of elongating and condensing spermatids. The transcripts coding for transition proteins, protamines, and TSSKs were reduced greatly inside the CBs due to the loss of phospho-GRTH, leading to the failure of chromatin remodeling, which is essential for the condensation of chromatin in developing spermatids during spermiogenesis. UPF2, which is involved in NMD, an mRNA surveillance pathway that eliminates transcripts with premature stop codons, was decreased in the CBs resulting in inefficient mRNA surveillance due to the loss of phospho-GRTH [10]. Different poly(A)-binding proteins (PABP) and Poly (Rc) binding proteins (PCBP) are found in the CB to support the role of the CB in mRNA processing, splicing, regulation, translation, and mRNA turnover. Due to the loss of phospho-GRTH, transcripts coding for these proteins were increased in CBs of GRTH KI mice, as decreased mRNAs in the CB require more stabilization from PABPC and PCBP proteins. Furthermore, transcripts of several initiation factors (*eIF4e2*, *eIF4ebp2*, *eIF3l* and *eIF3m*) together with mRNAs related to 60S subunit (Rpl10l/Rplp0) were increased and accumulated in the CB, resulting in the disruption of translation machinery of germ cells which caused spermatogenic arrest and loss of sperm in GRTH KI mice.

## 6. Conclusions

CBs are highly critical organelles in the developing sperm which control the spermatid elongation processes precisely by mediating the post-transcriptional and translational control. In the absence of phospho-GRTH there is a loss and degradation of mRNAs (in CBs) coding for essential proteins which are involved in chromatin condensation, spermatid elongation, and spermatozoa formation, resulting in a lack of sperm and infertility. Our studies in this line of work unraveled the role of phospho-GRTH on cell-specific regulation occurring in the CBs of germ cells. Additional functional analyses would reveal more specific mechanisms governing functional sperm production and male fertility. Furthermore, more studies will unravel the molecular shuttling events of mRNA transport to the CB and to the polysomes by phospho-GRTH, and its role in translation.
